# Extraperitoneal pediatric kidney transplantation of adult renal allograft using an en-bloc native liver and kidney mobilization technique

**DOI:** 10.1186/s12887-020-02422-0

**Published:** 2020-11-16

**Authors:** Mahmoud Alameddine, Joshua S. Jue, Mahmoud Morsi, Javier Gonzalez, Marissa Defreitas, Jayanthi J. Chandar, Jeffrey J. Gaynor, Gaetano Ciancio

**Affiliations:** 1 Department of Surgery and Urology, Miami Transplant Institute, University of Miami Miller School of Medicine, Jackson Memorial Hospital, 1801 N.W. 9th Ave, Suite 700, FL 33136 Miami, USA; 2 Department of Urology, Lenox Hill Hospital, Northwell Health, New York, NY USA; 3 Department of Urology, Hospital General Universitario Gregorio Marañón, Madrid, Spain; 4 Department of Pediatrics (Nephrology), Miami Transplant Institute, University of Miami Miller School of Medicine, Jackson Memorial Hospital, Miami, FL USA

**Keywords:** Pediatric kidney transplantation, surgical technique, extraperitoneal approach

## Abstract

**Background:**

We describe the safety and efficacy of performing pediatric kidney transplantation with a modified extraperitoneal approach that includes mobilization of the native liver and kidney.

**Methods:**

We retrospectively identified pediatric renal transplants performed using this technique between 2015 and 2019. Data on patient demographics, surgical technique, and intraoperative details were collected. Outcomes were measured by morbidity and re-operation at 90 days, as well as serum creatinine, allograft survival, and overall survival at 1 year.

**Results:**

Twenty-one patients with a median age of 5 (IQR 3–9) years, weighing 17.5 (IQR 14.5–24) kg were included. Median donor age was 24 (IQR 19–31) years. No intraoperative complications occurred. One child required a right native nephrectomy to allow sufficient space. Postoperatively, all patients had immediate graft function without urine leak or allograft thrombosis. 90-day morbidity and re-operation rates were zero. Both 1-year allograft and overall survival were 100% (on follow-up of all 21 patients through 1 year post-transplant), with a median serum creatinine of 0.58 (IQR 0.47–0.70) mg/dl at 1 year post-transplant.

**Conclusions:**

Pediatric kidney transplantation of adult renal allografts using an extraperitoneal approach with native liver and kidney mobilization has promising allograft and patient survival outcomes that eliminates peritoneal violation and may diminish the need for native nephrectomy.

## Background

Among the variety of treatments for end stage renal disease in the pediatric population, kidney transplantation has emerged as the best option for overcoming renal replacement therapy.[1] Long-term renal dialysis in children carries increased mortality rates, cognitive and learning impairment, and anemia.[2] Renal dialysis in children also exacerbates renal bone disease and leads to early epiphyseal closure.[3] Kidney transplantation has been shown to enhance quality of life and improve growth and development.[4] Innovations in immunosuppression and antiviral regimens have improved pediatric transplant outcomes that rival and even surpass those seen in adults.[5] The benefits of kidney transplantation are even present in children weighing under 15 kg.[6, 7] With these promising results, renal transplantation is recommended to be performed as soon as possible, even before dialysis is initiated.[3].

Historically, pediatric kidney transplantation has been performed within the peritoneum through a midline laparotomy to overcome the space limitations associated with transplanting a larger adult donor kidney into a small child.[8–11] This approach can be technically challenging, since many pediatric transplant recipients have had prior abdominal surgeries. Substantial morbidity also accompanies the intraperitoneal approach, which includes abdominal compartment syndrome (0.02%), early vascular compromise of the allograft (7.6%), and non-renovascular complications (5.3%).[9, 12, 13] To minimize these potential challenges and complications, an extraperitoneal approach was devised and has been performed in small children, where the allograft is transplanted in the retroperitoneal space without violation of the peritoneum.[11, 14, 15] In one study, extraperitoneal kidney transplantation in children resulted in earlier improvement in allograft function compared to the intraperitoneal approach.[16] Extraperitoneal kidney transplantation also facilitates easier allograft biopsy and peritoneal dialysis in pediatric patients who experience delayed graft function.

Proponents of the intraperitoneal approach have questioned the adequacy of surgical field exposure and allograft space with the extraperitoneal technique. The extraperitoneal approach has been criticized for the need to commonly perform a native nephrectomy in order to create sufficient space for renal allograft placement in small children. There is currently little data investigating techniques to create allograft space within the retroperitoneum.

We present a novel modification to the extraperitoneal approach that includes mobilization of the native liver and kidney to create ample space in the retroperitoneum and reduce the need for native nephrectomy. In this manuscript, we describe details of the operation and provide illustrations. The safety and efficacy of the technique are analyzed in 21 consecutive pediatric transplant recipients using both deceased and living donor adult allografts.

## Methods

We retrospectively reviewed all pediatric renal transplants performed at the Miami Transplant Institute from January 2015 to May 2019. Patients below the age of 13 years who underwent extraperitoneal kidney transplantation were identified. A single surgeon mobilized the liver and performed an en-bloc mobilization of the native kidney through a retroperitoneal approach when the size of the adult donor kidney was disproportionate to the recipient reception site. The demographic data of the recipients and donors are listed in Table 1. We evaluated warm and cold ischemia times, estimated blood loss, intraoperative complications, morbidity and re-operation at 90 days, as well as serum creatinine, allograft survival, and overall survival at 1 year.

**Table 1:** Patient demographics and clinical variables within the cohort

	Median (IQR)
Recipient age at transplant (years)	5 (3-9)
Recipient body weight (kg)	17.5 (14.5–24)
Donor age (years)	24 (19–31)
Recipient number of prior surgeries^a^	**Mode (range)** 2 (1–13)
	**N (%)**
Recipient gender	
Male	14 (66.6)
Female	7 (33.3)
Recipient history of bladder augmentation	6 (28.5)
Donor kidney type	
DD	14 (66.6)
LD	7 (33.3)

*DD* deceased donor; *LD* living donor, *IQR* interquartile range

^a^in 16 patients who had previous abdominal surgeries

All children received induction immunosuppression in the form of antithymocyte globulin 1 mg/kg (3 doses), basiliximab 10 mg (2 doses) and methylprednisolone starting at 10 mg/kg (maximum 500 mg/dose). Methylprednisolone was tapered by 2 mg/kg daily and discontinued by post-operative day 5 to 7, when a therapeutic level of tacrolimus was obtained (target level 6–8 ng/ml). All patients received a steroid-free maintenance regimen that included tacrolimus and mycophenolate mofetil. Oral tacrolimus was introduced when serum creatinine was < 3 mg/dl in children older than six years of age, and < 2 mg/dl in those who were younger. Oral mycophenolate mofetil was introduced on the second post-operative day at a dose of 600 mg /m^2^/ dose twice daily. Dose adjustment was performed according to white blood cell count and gastrointestinal tolerance. This study was approved by the Institutional Review Board at the University of Miami.

### Surgical Technique

**Fig. 1:**
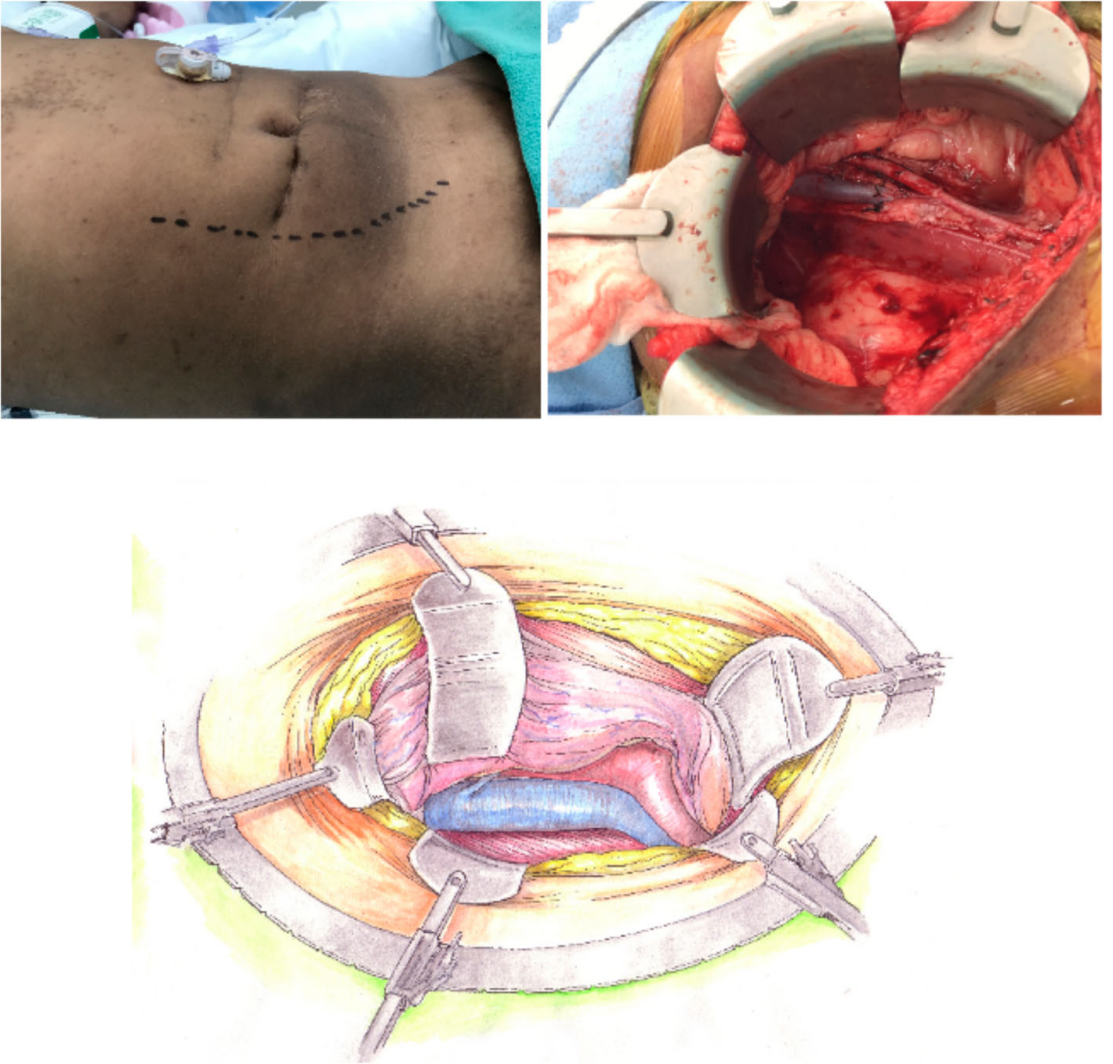
Gibson skin incision with retroperitoneal exposure of the inferior vena cava and the aorta

**Fig. 2:**
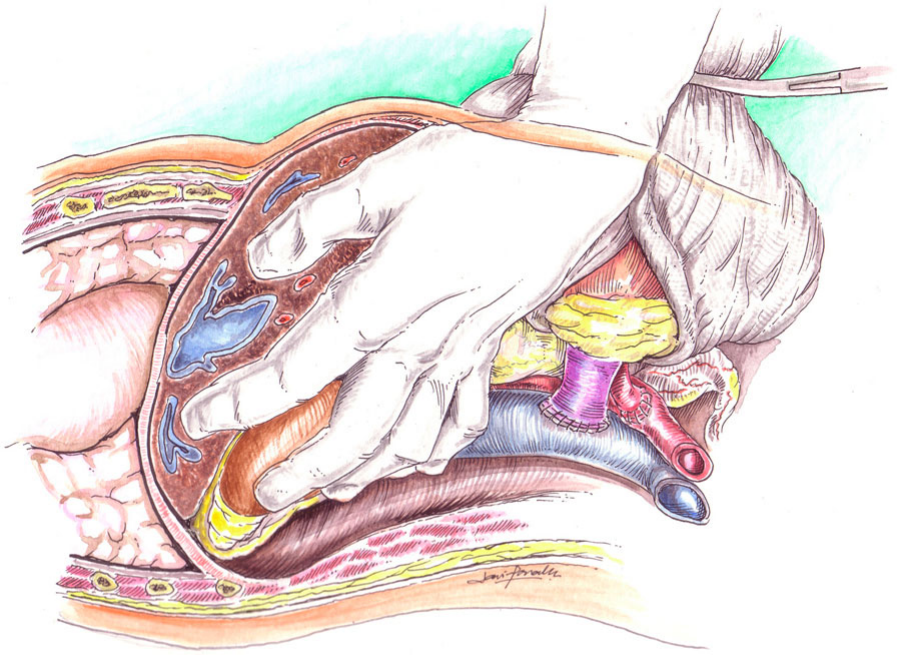
Anterior mobilization of the recipient liver and kidney through blunt dissection of the renal fossa. A sufficient space is created for the adult renal allograft

**Fig. 3:**
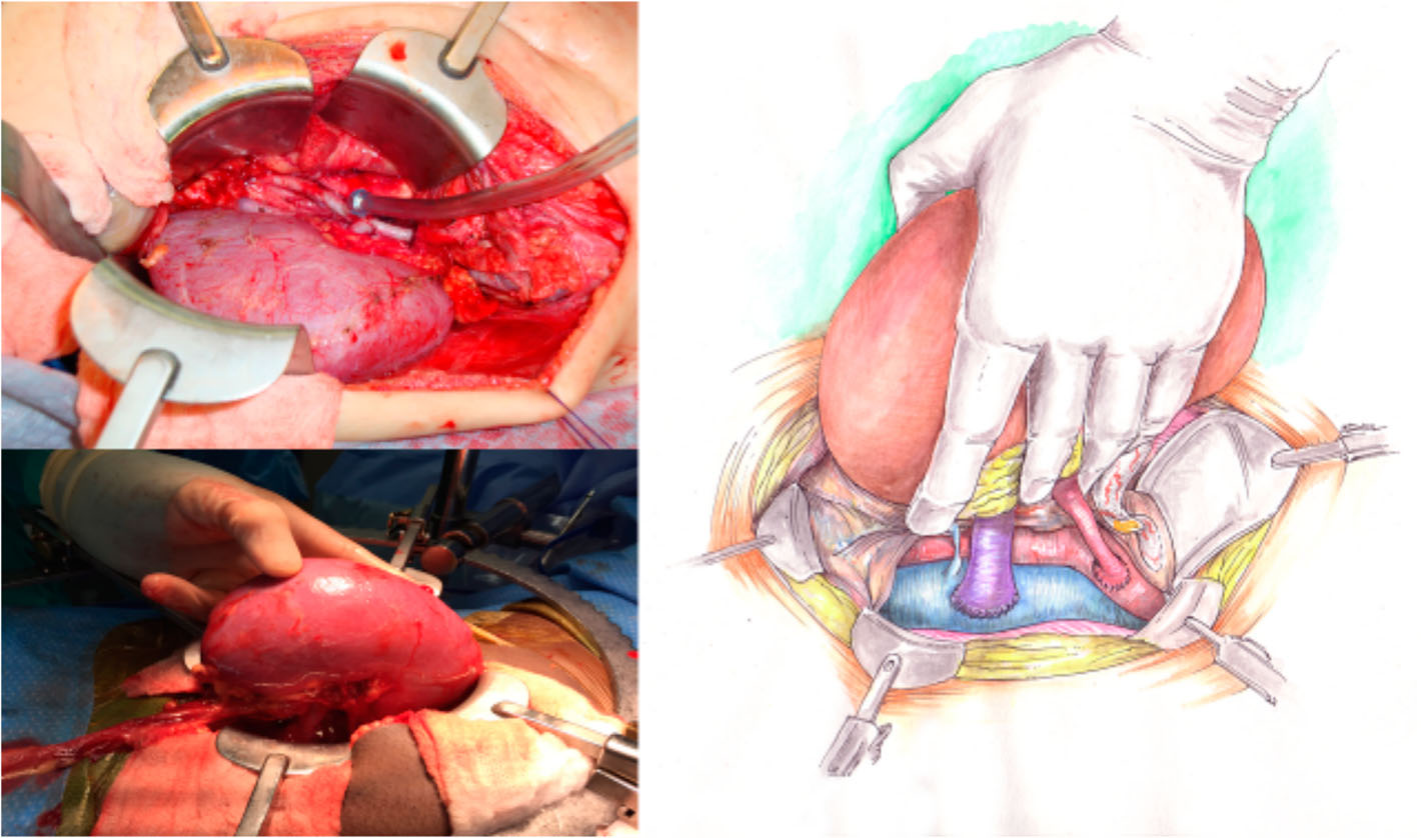
Allograft renal vein anastomosis to the IVC, and renal artery anastomosis to the right common iliac artery. The renal allograft is revascularized and hemostasis is attained

**Fig. 4:**
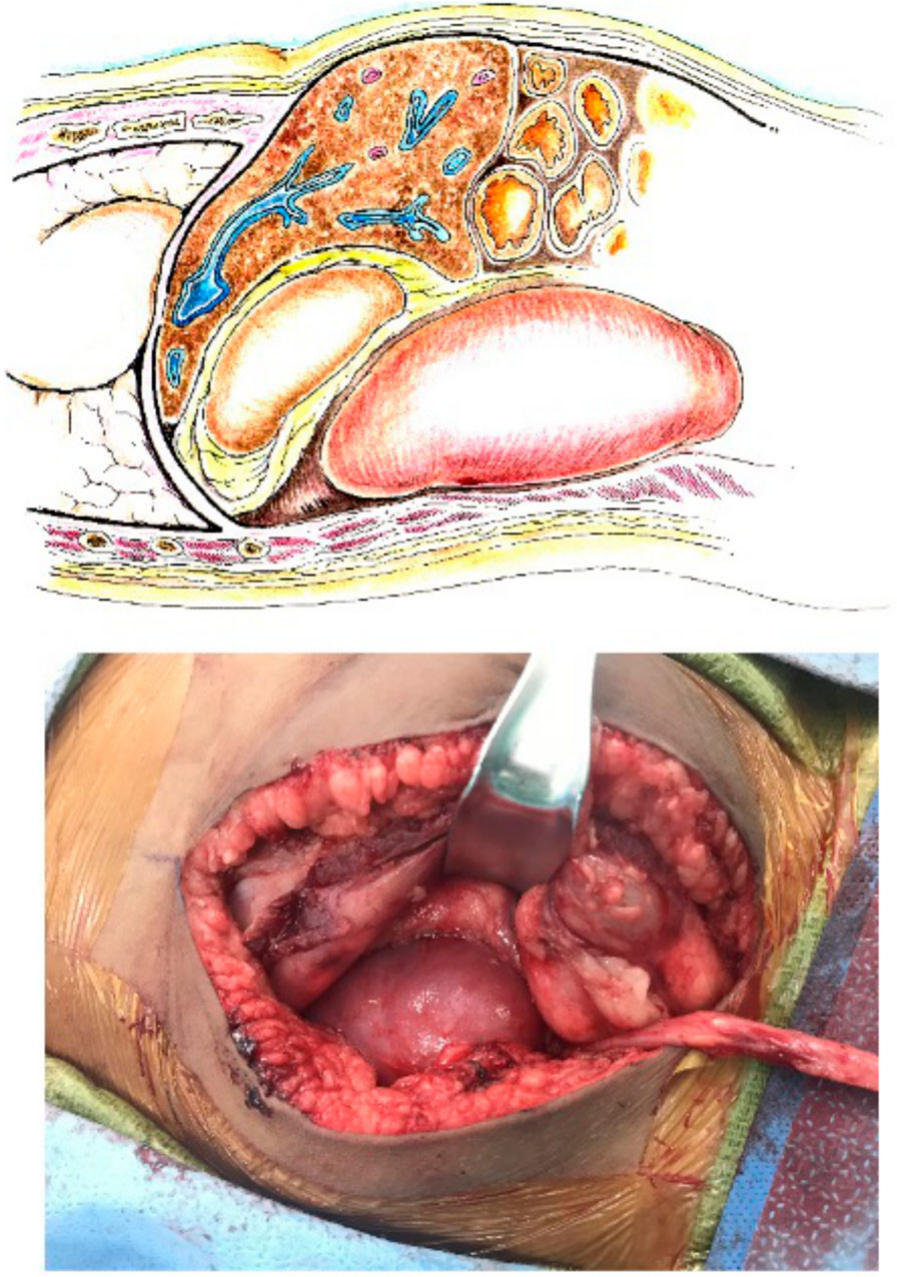
Adult renal allograft is placed in the retroperitoneal space

**Fig. 5:**
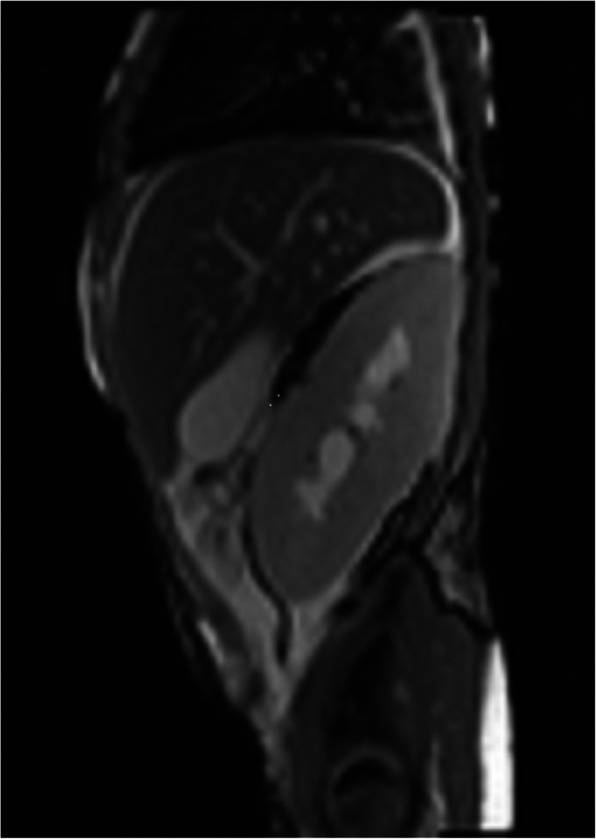
Sagittal section of an abdominal magnetic resonance imaging of a five-year old recipient with an adult renal allograft occupying about half of the abdominal space *All illustrations are freely available to use (not under copyrights). The drawings were made with ink and watercolor by co-author [JG]1

The operation begins with a Gibson incision that starts 2-finger breadths from the right pubic tubercle and can be extended to the costal margin, if necessary (Fig. 1). Allografts are placed on the patient’s right side to facilitate easier access to the major blood vessels. The abdominal wall is opened in layers until the peritoneum is encountered. The peritoneal membrane is reflected medially with gentle blunt dissection to avoid tears. Peritoneal tears should be repaired with 3 − 0 chromic continuous sutures. Blunt and sharp dissection of the right renal fossa is then performed to create a space posterior to the native kidney. Blunt dissection is facilitated by the surgeon’s hand within the para-renal fat between the posterior abdominal wall and Gerota’s fascia. The dissection is extended cephalad to mobilize and medially reflect the liver en-bloc with the native kidney to create a space for the new renal allograft (Fig. 2). This exposes the IVC, aorta, and right iliac vessels in the retroperitoneal space. A Bookwalter retractor is routinely used to assist and maintain this exposure. Lymphatics and lumbar veins are ligated to facilitate IVC mobilization. Once adequate exposure is obtained, the renal allograft is prepared on ice at the back-table. Venous and arterial control is attained using tangential clamps. Then, the renal allograft is brought onto the surgical field and placed in an anatomical position. The allograft renal vein is first anastomosed to the IVC using running 5 − 0 polypropylene sutures. The renal artery is then anastomosed to the aorta or right common iliac artery, depending on arterial size, using continuous 6 − 0 polypropylene sutures (Fig. 3). The allograft is then placed in the retroperitoneum posterior to the liver and native kidney (Fig. 4). The allograft ureter is connected via ureteroneocystostomy (a modification of the Lich-Gregoir technique) to the bladder. The same technique is applied if the bladder was previously augmented with small intestine, ureter, or sigmoid colon. Ureteral stent was not used in these cases. All patients were extubated after the transplant surgery without respiratory compromise. All patients received a nasogastric tube during the operation that was removed after extubation. We did not place any surgical drains at the end of the operation. Figure 5 depicts a sagittal view of abdominal magnetic resonance imaging for a five-year old recipient one year after the transplant of a large adult allograft. The allograft is retro-hepatic and occupies the space from the diaphragm to the bladder.

## Results

Twenty-one patients were retrospectively identified between January 2015 and May 2019, including 14 boys and seven girls. The median age of recipients was 5 (IQR 3–9) years, with seven children ≤ 3 years. The median body weight was 17.5 (IQR 14.5–24) kg, with seven children weighing ≤ 15 kg. Sixteen recipients had at least one previous abdominal surgery (mode 2, range 1–13). All donors were adults with a median donor age of 24 (IQR 19–31) years. Fourteen allografts were from deceased donors, and 7 were from living donors (Table 1). The primary causes of end stage renal disease are summarized in Table 2.

**Table 2:** Primary disease for the end stage renal failure

Diagnosis	Number of patients (n)	Percentage (%)
Obstructive uropathy	6	28.5
Prune belly syndrome	5	23.8
Kidney dysplasia	3	14.2
Hypoxia	2	9.5
VACTERL syndrome	1	4.7
Cystinosis	1	4.7
Wilms tumor	1	4.7
FSGS	1	4.7
Vasculitis	1	4.7

The allograft renal vein was anastomosed to the IVC in an end to side fashion in all 21 patients. The allograft renal artery was anastomosed to the aorta (2/21) or the right common iliac artery (19/21). The ureteroneocystostomy was most commonly performed to the bladder (15/21) but was also performed to the augmented bladder with small intestine (2/21), ureter (3/21), and sigmoid colon (1/21).

Median warm ischemia time was 30 (IQR 26–32) min, and median cold ischemia time was 18 (IQR 12–30) hours. The median estimated blood loss was 20 (IQR 20–30) ml (Table 3). Most patients received transfused packed red blood cells before finishing the vascular anastomosis, in order to compensate for the larger vascular volume of the donor renal allograft and to maintain appropriate blood pressure during kidney reperfusion. No intraoperative complications occurred. The operation failed to achieve an adequate space in one patient, which resulted in a right native nephrectomy. Two patients previously received a bilateral nephrectomy for Wilm’s tumor and recurrent urinary tract infections, respectively. In these two cases, the transplant operation consisted of retroperitoneal mobilization of the liver to create sufficient place for the adult allograft. Postoperatively, all patients exhibited immediate graft function without urine leak, ureteral stenosis, or allograft thrombosis. The 90-day morbidity and re-operation rates were 4.8% (1/21) and 0.0% (0/21), respectively. No abdominal complications such as ileus or bowel obstruction were observed. One year post-transplant follow-up was complete for all 21 patients. Both 1-year allograft and overall survival rates were 100%, with a median serum creatinine of 0.58 (IQR 0.47–0.70) mg/dl. No native kidney problems were encountered at the most recent follow-up among all patients.

**Table 3:** Summary of outcomes

	Median (IQR)
WIT (min) CIT (hours)	30 (26–32) 18 (12–30)
EBL (mL)	20 (20–30)
Serum Cr at 1 year (mg/dL)	0.58 (0.47–0.70)
	**N (%)**
Intraoperative complications	0 (0)
Morbidity at 90 days^a^ Re-operation at 90 days	1 (4.8) 0 (0)
Allograft survival at 1 year	21 (100)
Overall survival at 1 year	21 (100)

*WIT* warm ischemia time; *CIT* cold ischemia time; *EBL* estimated blood loss; *Cr* creatinine, *IQR* interquartile range

^a^BK viremia

## Discussion

While an extraperitoneal approach to kidney transplantation in the pediatric population has previously been described, this manuscript is the first to describe and present outcomes when using this technique with en-bloc mobilization of the native liver and kidney. Advantages of this technique begin with the J-shaped external pararectal incision, which avoids muscle splitting and results in less postoperative pain. The extraperitoneal dissection preserves peritoneal integrity to minimize the morbidities associated with intraabdominal operations, especially in children with extensive surgical histories. Intraabdominal complications after intraperitoneal pediatric kidney transplantation can be categorized as gastrointestinal, fluid collections, urogenital, and vascular. Gastrointestinal complications were the most common in a series of 146 patients.[17] Bowel obstruction is the most common gastrointestinal complication, but incisional hernia, volvulus, and abdominal compartment syndrome can also occur, especially in those who receive large grafts.[9, 12, 17] On multivariable analysis, prior abdominal surgery was found to be a significant risk factor in developing a complication.[17] In our cohort, most of the children (16/21) had major prior abdominal surgeries. One patient in our cohort had 13 prior abdominal surgeries, primarily for reconstruction of congenital anomalies. The en-bloc native liver and kidney mobilization technique utilizes an extraperitoneal approach to eliminate major gastrointestinal complications.

Nephrectomy of the native kidney is commonly performed when utilizing the extraperitoneal approach in pediatric recipients due to size mismatch between the adult renal allograft and the surgically created space. Native nephrectomy has been reported to increase the risk of operative complications, including longer operating time, requirement for blood transfusions and prolonged hospital stay.[18] In a series of 46 children who underwent transplantation through an extraperitoneal approach, eight nephrectomies, two nephroureterectomies, and one ureterectomy were performed.[14] A similar finding of 4 nephrectomies was reported in an extraperitoneal pediatric kidney transplant series of 29 patients (Table 4).[11] In our study, mobilization of the native liver and kidney successfully created a sufficient space in the retroperitoneum for the adult allograft in all but one patient.

**Table 4:** Series of extraperitoneal approach in children and number of native nephrectomies

Reference	Mobilization of the native organs	Number of patients (n)	Recipient size (mean, Kg)	Donor (adult/pediatric)	Number of native nephrectomies
Fangmann et al. 1996 [19]	Mobilization of the retro-hepatic area	8	11.4 ± 2	Adult	Performed when indicated but number of cases was not reported
Nahas et al. 2000 [14]	none	46	16.6 (range 8.3–20)	Adult and pediatric	10
Furness PD et al. 2001 [11]	none	29	11.2 (range 8-14.8)	Not reported	4
Vitola SP et al. 2013 [7]	none	62	12.3 ± 2.1	Adult and pediatric	0
Gander R et al. 2017 [6]	none	42 EP 2 IP (LKT)	10.10 ± 2.9	Pediatric	4

*EP* extraperitoneal; *IP* intraperitoneal; *LKT* liver-kidney transplant

There is scarce literature describing extraperitoneal techniques that create allograft space utilizing the mobilization of native organs. Fangmann et al. report a series of 8 children who underwent extraperitoneal placement of living related adult renal allografts.[19] The author briefly reported performing partial right liver mobilization to place the allograft in the retro-hepatic area when there was size mismatch between the donor kidney and recipient. The number of patients who underwent this mobilization technique was not reported. Ultimately, nephrectomy was performed when native kidneys were large, and one child suffered delayed wound closure.[19].

En-bloc liver and kidney mobilization facilitates adequate exposure of the great vessels. Mobilization also reduces tension exerted on the venous anastomosis between the renal allograft and IVC, which may help avoid impairment of venous drainage and kinking. Extraperitoneal placement of the renal allograft also theoretically limits allograft mobility, which may protect the vascular and ureteral bed. Placement of the renal allograft freely in the peritoneum may increase its potential for movement, which theoretically increases the risk of vascular or ureteral compression or torsion.[13].

Ultimately, the native liver and kidney mobilization technique creates more clinically significant space compared to other extraperitoneal approaches, while carrying complication rates that are similar or improved to those reported in intraperitoneal and extraperitoneal series.[11, 13, 14, 17, 20]. Our study is primarily limited by its retrospective nature and relatively small number of patients. Our study also lacks the inclusion of a control group that did not undergo extraperitoneal mobilization. Although this study included patients with a median weight of 17.5 kg, future studies should investigate the applicability of this approach in even smaller recipients. While mobilization of the native liver and kidney has reduced the need for nephrectomy to create additional allograft space, nephrectomy may still be indicated to treat certain conditions such as nephrotic syndrome, kidney tumor, intractable hypertension, and recurrent pyelonephritis secondary to reflux.

## Conclusions

In conclusion, kidney transplantation in children via an extraperitoneal approach with native liver and kidney mobilization creates adequate retroperitoneal space for adult graft placement. This technique eliminates peritoneal violation and may diminish the need for native nephrectomy. Overall, this surgical technique is a safe and effective approach to kidney transplantation that may increase the donor pool in the pediatric population.

## Data Availability

The datasets used and analysed during the current study are available from the corresponding author on reasonable request.

## Abbreviations

IVC: Inferior vena cava
